# The Role of Exosome-miRNA as Biomarkers of Alzheimer’s Disease: A Systematic Review of Case-Control and Longitudinal Studies

**DOI:** 10.62641/aep.v54i2.2109

**Published:** 2026-04-15

**Authors:** Haiqing Zhou, Ying Liu, Kaijun Li, Shangdong Mou, Xia Cao, Qingjuan Chen

**Affiliations:** ^1^Department of Infection Prevention and Control, 3201 Hospital, 723000 Hanzhong, Shaanxi, China; ^2^Department of Oncology, 3201 Hospital, 723000 Hanzhong, Shaanxi, China; ^3^Department of Oncology, The Zhenba County People’s Hospital, 723600 Hanzhong, Shaanxi, China

**Keywords:** Alzheimer’s disease, exosomes, biomarkers, neurodegenerative diseases

## Abstract

Alzheimer’s disease (AD) is a progressive neurodegenerative disorder, and its diagnosis remains challenging. Exosomal microRNAs (miRNAs), due to their stability, tissue specificity, and ability to cross the blood–brain barrier, show significant promise as ideal biomarkers for AD. The present study aimed to systematically elucidate the relationship between the exosomal miRNA expression changes and AD pathogenesis (including Aβ deposition, Tau protein phosphorylation, and neuroinflammation) and to evaluate their potential utility in clinical screening and therapeutic interventions. A systematic literature search was conducted in the PubMed and Web of Science databases to identify human case–control or cohort studies reporting expressions of mature exosomal miRNAs in the serum, plasma, cerebrospinal fluid, saliva, or central nervous system cells. After evaluating the quality of the studies using the National Institutes of Health quality assessment tool, the identified differentially expressed miRNAs were summarized and functionally integrated. Among the 390 screened records, 48 studies (n = 3046 AD patients) met the inclusion criteria. The analysis identified 120 exosomal miRNAs that are differentially expressed at different AD ages. Six miRNAs (miR-125b, miR-146a, miR-193b, miR-185-5p, miR-29b/c, miR-21-5p) were most consistently reported and showed significant correlation with AD pathology.

## Introduction

Alzheimer’s disease (AD) is an age-related, progressive neurodegenerative disorder. Pathologically, it is characterized by the accumulation of senile plaques and neurofibrillary tangles in the brain [1]. Clinically, it manifests as cognitive impairments, progressive memory loss, and behavioral alterations [2]. Epidemiological data suggest that over 50 million individuals are affected by AD [3]. As the global population ages, the incidence of AD is increasing annually [4], placing a substantial burden on patients, caregivers, and society [5]. Presently, the diagnosis of AD primarily depends on the criteria established by the National Institute on Aging and Alzheimer’s Association (NIA-AA) [6]. In 2018, the NIA-AA introduced the “AT(N)” diagnostic framework, which incorporates Aβ, Tau, and other biomarkers, alongside traditional clinical symptoms, neuroimaging findings, and cognitive assessments [7, 8]. In 2020, the criteria were further updated to the “ATNIVS” framework, which additionally included “I” (inflammatory mechanisms), “V” (vascular brain injury), and “S” (α-synuclein) [9]. This revision underscores the increasing significance of biomarkers in AD diagnosis.

Exosomes are lipid bilayer–enclosed vesicles derived from endosomes, typically measuring 30–150 nm in diameter [10]. They are secreted into biological fluids—including serum, plasma, urine, saliva, cerebrospinal fluid, and amniotic fluid—by diverse cell types such as neurons as well as glial, immune, and mesenchymal stem cells (MSCs) [11]. The major bioactive components of exosomes include proteins, lipids, and microRNAs (miRNAs). After being synthesized and released by donor cells, exosomes deliver their contents into the cyto-plasm of target cells via indirect binding to signaling receptors or direct fusion with the plasma mem-brane, thereby mediating intercellular communication and regulating biological functions [12, 13, 14]. MiRNAs are non-coding, single-stranded RNA molecules encoded by endogenous genes, generally comprising approximately 22 nucleotides [15]. They regulate gene expression by binding to the 3^′^ UTR of target mRNAs and participate in processes including cell proliferation, differentiation, development, and metabolism [16]. Early studies reported that miRNAs contribute to AD pathogenesis by protecting neurons, preserving synaptic plasticity, regulating Aβ production, and modulating Tau protein phosphorylation [17, 18, 19, 20, 21]. However, circulating miRNAs in the blood are readily degraded by RNases, making stable detection challenging, and they lack tissue specificity [22, 23], which limit their clinical diagnostic utility. In the central nervous system (CNS), exosomal miRNAs are secreted by diverse cell types, including neurons and glial cells. The phospholipid bilayer of exosomes protects miRNAs from RNase degradation, thereby maintaining their stability in blood and tissues. Furthermore, they can cross the blood–brain barrier (BBB), enabling delivery to diseased brain regions where they exert therapeutic effects [24, 25]. Thus, exosomes stabilize the biomarkers and offer substantial potential for diagnosis, mechanistic studies, and targeted therapies in neurodegenerative disorders, including AD [11, 26]. The present study aimed to comprehensively review existing research on alterations in exosomal miRNA expression and their functional roles in AD and to further explore specific miRNAs related to the pathophysiology of AD, thereby providing insights for clinical screening and preventive interventions.

The present study aimed to systematically elucidate the relationship between exosomal miRNA expression alterations and AD pathogenesis and to assess their potential utility in clinical screening and therapeutic interventions.

## Materials and Methods

### Literature Search Strategy

We conducted a systematic literature search in the PubMed database. The following search terms were utilized: Alzheimer, AD, amyloid β, exosome, and microRNAs, miRNA, or miRNAs. The specific search strategy was (“Alzheimer” [All Fields] OR “Alzheimer’s disease” [MeSH Terms] OR “AD” [All Fields]) AND “exosome” [All Fields] AND (“microRNAs” [MeSH Terms] OR “microRNAs” [All Fields] OR “miRNA” [All Fields] OR “miRNAs” [All Fields]) NOT (‘review’ [Publication Type] NOT “review literature as topic” [MeSH Terms] NOT “review” [All Fields]). The search time limit was set from the database creation date to the latest available data. The retrieval time was until July 8, 2025.

### Inclusion and Exclusion Criteria 

#### Inclusion Criteria 

Study Type: Original research studies (including human case-control studies and cohort studies); Study Population: Adult patients, including both the AD group and non-AD control group; Detection Target: Changes in the expression of mature miRNAs derived from exosomes; Sample Source: Tissue or bodily fluids, such as serum and cerebrospinal fluid; Language Restriction: Only full-text literature published in English was included.

#### Exclusion Criteria 

We excluded non-original studies, including reviews, conference abstracts, commentaries, editorials, and technical reports, secondary analyses based on prior studies or public databases, studies involving patient populations receiving pharmacological interventions, and literature for which the full text was unavailable.

#### Literature Screening Process

Duplicate literature was removed using literature management software (e.g., EndNote), and two researchers independently conducted an initial review of titles and abstracts to exclude studies not meeting the inclusion criteria. Full-text articles of the remaining studies were obtained, and the same researchers performed a re-screening based on the predefined inclusion and exclusion criteria. In case of disagreements, a consensus was reached through discussions and negotiations. If consensus could not be reached, a third researcher was consulted to make the final decision.

#### Data Extraction and Integration

**Fig. 1.**
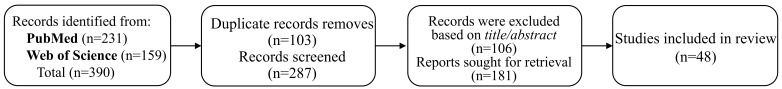
**The Flowchart of inclusion and exclusion criteria in the search strategy**.

Basic information extraction: The authors, publication year, study design, sample source, sample size, and detection techniques of the included studies were recorded. Differential expression analysis: Exosomal miRNAs showing significant changes in expressions (*p* < 0.05) between groups were extracted and annotated up or downregulated. Data integration: Exosomal miRNAs reported in more than three studies were screened, and their biological functions related to the pathological mechanisms of AD were summarized. Owing to the substantial heterogeneity across studies, we only conducted a qualitative (narrative) synthesis. The inclusion and exclusion criteria during the specific retrieval process are shown in Fig. 1.

#### Research Quality Assessment

The present study used the National Institutes of Health quality assessment tool to evaluate the methodological quality of the included case–control studies. The tool contains 12 binary items, which address key methodological areas, including study population, sample size, inclusion/exclusion criteria, and statistical analysis, assessing the potential risk of bias. The evaluation options for each item are “Yes”, “No”, “Cannot Determine”, “Not Applicable”, or “Not Reported”. If a criterion is fully met in a study, the item is scored one point, with a theoretical total score of 12; a higher score indicates a higher study quality. This tool does not set a uniform classification threshold; thus, two reviewers independently scored each study based on the specific objectives and characteristics of this systematic review. In the case of disagreements, a consensus was first sought through a discussion; if unresolved, a third reviewer was consulted to ensure a consistent and objective evaluation process.

## Results

### Literature Screening and Quality Assessment

According to the system retrieval criteria, 231 and 159 records were identified through the PubMed and Web of Science databases, respectively. After screening the titles and abstracts of 390 records, a total of 48 papers met the inclusion criteria, with 3046 patients included in the present systematic review. The inclusion criteria for selecting the AD patients were as follows: only individuals with a confirmed diagnosis of AD, dementia of the Alzheimer’s type (DAT), or mild cognitive impairment (MCI) that progressed to AD were included. Animal models, healthy controls, and patients with other neurodegenerative diseases, such as frontotemporal dementia or Parkinson’s disease, were excluded from the analysis.

Altogether, 3046 AD patients were enrolled, including partially overlapping cohorts. The AD stage distribution was as follows: subjective cognitive decline (SCD), ≥241 cases; MCI, ≥1114 cases; and clinical AD/DAT, ≥1691 cases. The included studies reported on disease progression from the preclinical stage (SCD/MCI) to DAT. Of these, 38 were case–control studies (AD vs. healthy controls), and 10 were longitudinal studies that conducted an exosomal miRNA analysis.

### Tissue Sources of Exosomes in Alzheimer’s Disease Patients

In the 48 studies included in the final analysis, the tissue sources of exosomal miRNAs were categorized into the following three main types: human body fluids, CNS, and engineered cells (Table 1). Among the clinical samples obtained from human body fluids, 28 studies mentioned exosomal miRNAs derived from the serum/plasma. This method is non-invasive and easily accessible, making it the most clinically practical, although it contains exosomes from multiple cell types throughout the body. Exosomes derived from saliva were reported in two studies; saliva collection is completely non-invasive and suitable for AD screening. Only one study reported exosomes collected from urine, which may exhibit a weaker correlation with AD. The CNS cells include the microglia (10 articles), astrocytes (five articles), neurons (eight articles), and choroid plexus cells (two articles), which secrete exosomes that are released into the interstitial fluid of the brain tissue and then enter into the CSF through diffusion or active transport, also considered to originate from the CSF. Although CSF exosomes directly reflect the pathological state of the CNS and offer higher pathological specificity, their collection is moderately invasive. Engineered or stem cell-derived exosomes, including those from the MSCs (nine studies), adipose-derived stem cells (three studies) and dendritic cells (two studies), were primarily used in AD treatment research.

**Table 1.** **Classification and research overview of exosome origins**.

Source category	Sample/Cell type	Number of studies (n)	Notes
Body fluid	Serum/plasma, saliva, urine.	31	Main sources for exosome research.
CNS cells	Microglia, neurons, astrocytes, neurons, choroid plexus cells.	33	Mostly used in studies of neuroinflammatory mechanisms.
Engineered cells	Mesenchymal stem cells, adipose-derived stem cells, genetically modified cells.	14	Commonly used in therapeutic delivery research.

Note: 27 studies analyzed exosomes from multiple sources simultaneously.

### Types and Expression Differences of Exosomal miRNAs in Alzheimer’s Disease

A comprehensive analysis of data from 48 studies involving AD patients and controls identified 120 differentially expressed exosomal miRNAs. Among these, the following six exosomal miRNAs were reported with a high frequency (four or more occurrences) in AD patients: miR-125b (sevenoccurrences), miR-146a (six occurrences), miR-193b (five occurrences), miR-185-5p (four occurrences), miR-29b/c (four occurrences), and miR-21-5p (four occurrences). Compared with the controls, three of these high-frequency miRNAs—miR-125b, miR-193b, and miR-185-5p—were consistently downregulated in the serum or neurons of AD patient. Low serum levels of miR-125b contribute to the clinical diagnosis of AD. The remaining three miRNAs—miR-146a, miR-29b/c, and miR-21-5p—displayed inconsistent expression patterns. In patients with MCI, the plasma exosomal miR-483-5p [area under the curve (AUC) = 0.901] remained highly expressed and may serve as a biomarker for MCI [27]. In AD patients, salivary exosomal miR-485-3p was closely associated with cerebral Aβ deposition and may serve as a marker for this pathology [28].

### The Role of Differentially Expressed miRNAs in Alzheimer’s Disease

**Fig. 2.**
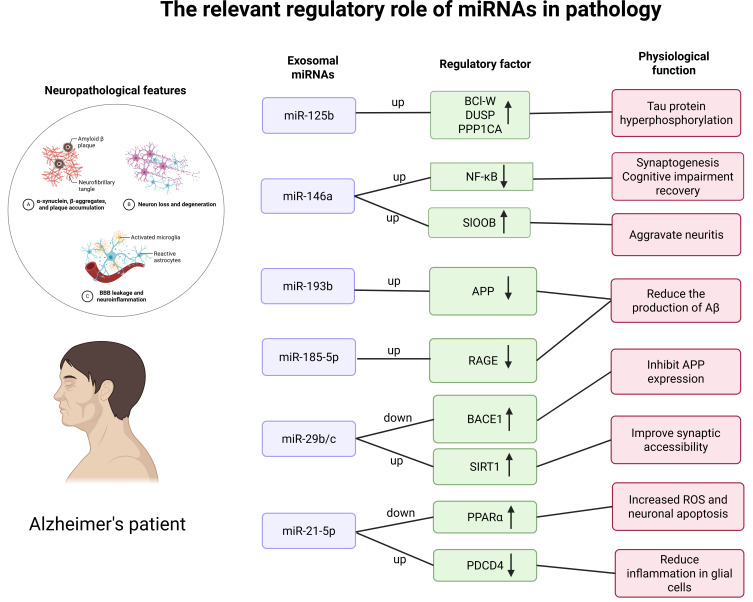
**The frequently altered miRNAs in Alzheimer’s disease research and their associated roles in the pathophysiology**. ↑, up-regulated; ↓, down-regulated.

A systematic analysis of 48 studies revealed that exosomal miRNAs are involved in multiple pathological processes of AD via diverse pathways. These include pathological protein formation (miR-193b, miR-185-5p, miR-125b), neuroinflammation (miR-146a, miR-21-5p, miR-233), neuronal regeneration and synaptic structure (miR-132, miR-124, miR-135a), gut microbiota–exosomal miRNA interactions (miR-3120-3p, miR-6529-5p, miR-124-3p), epigenetic modification (miR-29b, miR-132, miR-124-3p), mitochondrial function repair (miR-146a, miR-485-3p, miR-21), maintenance of BBB integrity (miR-155, miR-126-3p), pathological protein transcellular diffusion (miR-125b, miR-21), and biomarker potential for AD diagnosis (miR-483-5p, miR-455-3p, miR-125b), among others. Table 2 (Ref. [24, 29, 30, 31, 32, 33, 34, 35, 36, 37, 38, 39, 40, 41, 42, 43, 44, 45]) summarizes the role of these frequently occurring miRNAs. Based on the abovementioned screening process, we identified multiple exosomal miRNAs associated with AD pathology. These miRNAs participate in critical processes, including Aβ production, Tau protein phosphorylation, and neuroinflammation by regulating specific targets. The overall regulatory network of these miRNAs is shown in Fig. 2.

**Table 2.** **The roles of frequently occurring miRNAs in the included studies**.

miRNAs	Category of role	Determined role in individual study
miR-125b	Diagnosis marker	∙ miR-125b shows its sensitivity and specificity in diagnosing AD [29].
	The driving factors of AD core pathology	∙ miR-125b was positively correlated with cognitive function [30].
		∙ miR-125b was involved in the pathological process of AD through tau protein hyperphosphorylation [31].
miR-146a	Diagnosis marker	∙ miR-146a is significantly upregulated in the brain tissue and bodily fluids of AD patients [32].
	Epigenetic regulation	∙ miR-146a mediates synaptic dysfunction, mitochondrial dysfunction, and neuronal death by targeting mRNAs encoding synaptic-associated proteins, mitochondria-associated proteins, and membrane proteins [33, 34, 35].
	Treatment marker	∙ miR-146a can reduce neuroinflammation and act as a protective agent for neurons [36].
	Dual regulatory role	∙ miR-146a had the characteristics of a “patho-protection” dual role [37, 38].
miR-193b	AD regulators	∙ miR-193b reduced the production of β-amyloid (Aβ) by inhibiting the expression of amyloid precursor protein (APP) and regulated the AD-related pathological processes [39].
	Diagnosis marker	∙ The expression level of miR-193b varies in AD patients [24].
miR-185-5p	AD early intervention target	∙ miR-185-5p activated the PI3K/Akt signaling pathway, inhibited neuronal apoptosis, and improved cell survival [40].
	Treatment marker	∙ miR-185-5p was significantly negatively correlated with the Aβ42/Aβ40 ratio of cerebrospinal fluid [41].
		∙ miR-185-5p was negatively correlated with hippocampal Aβ plaques [42].
miR-29b/c	Dual regulatory role	∙ miR-29b/c exerted a dual regulatory role by targeting amyloid production and mitochondrial pathways in AD [43].
miRNAs	Category of role	∙ Determined role in individual study
miR-21-5p	Dual regulatory role	∙ Downregulation of miR-21-5p in neuron-derived exosomes promoted neuroinflammation and apoptosis [37, 44].
	Multifunctional regulator	∙ When delivered by MSC exosomes, miR-21-5p inhibits inflammation and promotes protection [38, 45]. miR-21-5p played different roles in different pathological stages of AD [37].

## Discussion

This study provides a systematic review of the current literature examining alterations in exosomal miRNA expressions in the pathophysiology of AD. Altogether, 120 differentially expressed miRNAs were identified from 48 studies on AD; however, many were reported only once or twice, limiting the ability to fully elucidate their physiological functions. To better clarify the role of exosomal miRNAs in AD pathology, our analysis was focused on the following six most frequently reported species: miR-125b, miR-146a, miR-193b, miR-185-5p, miR-29b/c, and miR-21-5p. Among them, miR-125b, miR-146a, and miR-21 are among the most extensively investigated miRNAs in biological fluids and biopsy specimens from AD patients [46, 47]. Xian Duan and colleagues [48] reported a considerable upregulation of miR-125b-1-3p expression in plasma-derived exosomes obtained from AD patients, demonstrating a sensitivity of 82.1% and a specificity of 67.7%. Conversely, another study has observed a marked downregulation of miR-125b levels in the serum of patients with AD compared to that of healthy controls, with corresponding sensitivity and specificity values of 80.8% and 68.3%, respectively [29]. Moreover, the expression levels of exosomal miR-125b derived from the serum/plasma indeed showed differential changes [43, 44, 49]. This may be caused by the differences in exosome isolation and detection techniques across different research platforms. However, in CSF, the exosomal miR-125b-5p levels in early- and late-onset AD patients are higher than those in the control group [50, 51], which may be related to the BBB, CNS microenvironment, and origin of exosomal miRNAs.

It is speculated that existing studies generally lack distinctions in disease staging for AD patients and that exosomal miRNAs exhibit different expression patterns at various stages of AD development. This variation in expression suggests that the key miRNA functions depend on the tissue or cell source of the exosomes. Additionally, research on the role of miR-125b in the pathological mechanisms of AD has mainly focused on animal experiments. In APP/PS1 transgenic mice, the miR-125b expression is positively correlated with cognitive function [30]. Injecting miR-125b into the hippocampus of mice reportedly impairs their associative learning ability, accompanied by Bcl-W, DUSP, and PPP1CA downregulation, leading to increased Tau protein phosphorylation [31]. This indicates that miR-125b participates in the pathological process of AD through excessive Tau protein phosphorylation. In summary, serum/plasma-derived exosomal miR‑125b‑1‑3p and total serum miR‑125b exhibit sensitivity and specificity for distinguishing AD patients from healthy controls in existing case–control studies. This suggests their potential use for the non-invasive diagnosis of AD in the early stages. Meanwhile, the specific increase in CNS-derived exosomal miR-125b levels may contribute directly to the underlying pathology of AD. However, their diagnostic performance is influenced exosome isolation methods, detection platforms, and disease stage, and further validation in larger sample sizes and standardized multicenter studies is needed.

Exosomal miR-146a is the second most frequently detected miRNA in AD patients. Altered miR-146a expression has been observed in AD patients and can be quantified in various bodily fluids, including plasma, serum, and CSF [35]. A previous study has found a specific upregulation of miR-146a in the brain tissues of AD patients and discovered its association with the downregulation of complement factor H, suggesting that the changes in miR-146a levels are a sensitive indicator of the inflammatory microenvironment in AD [32]. miR-146a reportedly mediates synaptic and mitochondrial dysfunction, as well as neuronal death, by targeting mRNAs encoding proteins associated with synapses, mitochondria, membranes, and other cellular components [33, 34, 35]. Chronic brain inflammation is a hallmark of neurodegenerative diseases and considerably influences disease onset and progression, such as in AD [52]. miR-146a is transcriptionally regulated by NF-κB [53]. It has been demonstrated that enhanced miR-146a secretion in mouse choroid plexus-derived exosomes reduces astrocyte inflammation, increases synaptic density in the hippocampal subiculum, and prevents cognitive dysfunction in AD model mice [5]. Evidence further indicates that miR-146a can reverse astrocyte and microglial polarization, attenuate neuroinflammation, and promote oligodendrocyte precursor cell differentiation, thereby preserving normal myelin function [36]. These findings indicate that CNS-derived exosomal miR-146a exerts neuroprotective effects by reducing glial cell inflammation. The therapeutic potential is corroborated by studies using stem cell-derived exosomes as delivery vehicles. For instance, miR-146a secreted by bone marrow-derived MSCs is taken up by astrocytes, where it reduces NF-κB expression, restores astrocyte function, promotes synapse formation, and improves cognitive deficits [54]. Although miR-146a expression increases in the brain [55], this elevation does not persist throughout the entire course of AD pathology [56]. Nevertheless, CNS-derived exosomal miR-146a levels are considerably reduced in the CSF of AD patients [57]. Moreover, miR-146a is markedly downregulated in microglia-derived exosomes, which inhibit the NF-κB pathway by targeting TRAF6 and IRAK1, thereby alleviating neuroinflammation and improving cognitive function [37]. Conversely, miR-146a expression is upregulated in astrocyte-derived exosomes, exacerbating neuroinflammation by promoting the release of S100B and IL-1β [38]. This bidirectional regulatory mechanism suggests that miR-146a functions as a pathological-protective “double-edged sword”, exerting anti-inflammatory effects under physiological conditions while exacerbating inflammatory damage under pathological states. These findings also highlight the potential of miR-146a as a diagnostic biomarker for early detection and disease progression monitoring in AD patients. Future research should aim to develop cell-specific delivery systems capable of precisely modulating their function.

Exosomal miR-193b levels have been consistently reported to be downregulated in the serum and CSF [39, 58]. Compared with controls, plasma exosomal miR-193b levels are reduced in patients with MCI and DAT [59]. APP plays a pivotal role in AD pathogenesis. Under normal physiological conditions, miR-193b binds to the 3^′^ UTR of APP and inhibits its translation. In AD, exosomal miR-193b is consistently downregulated, which removes the miRNA-mediated repression on APP mRNA, increases the APP protein levels, and ultimately elevates Aβ production [39]. Additionally, the downstream direct target of miR-193b, β-site amyloid precursor protein cleaving enzyme 1 (BACE1), can inhibit β-secretase activity and reduce Aβ fragment production [60]. The target LRP1 facilitates Aβ clearance across the BBB, increasing transport efficiency by approximately 50%. In APP/PS1 transgenic mice, hippocampal miR-193b expression is downregulated, and its target GSK3β indirectly inhibits Tau hyperphosphorylation [59]. Furthermore, ABCA1-labeled exosomes in the serum of AD patients contain elevated miR-193b levels [61]. In summary, these data indicate that miR-193b downregulation occurs early, correlates with upstream APP/Aβ dysregulation and downstream Tau hyper-phosphorylation, and can be robustly quantified in both the CSF and exosome-enriched serum. Accordingly, Zhou *et al*. [24] developed a dumbbell-shaped aptamer sensor that accurately differentiated early-stage AD patients from non-AD controls, thereby confirming the diagnostic potential of miR-193b. In future therapeutic strategies, engineered stem cell-derived exosomes overexpressing miR-193b could be employed as delivery vehicles to treat AD, thereby effectively reducing cerebral Aβ production in patients.

Exosomal miR-185-5p has been identified as a highly expressed miRNA in AD patients. In these patients, miR-185-5p expression is consistently downregulated in both the serum and neuron-derived exosomes [40, 62]. The serum exosomal miR-185-5p levels are significantly negatively correlated with the CSF Aβ42/Aβ40 ratio 243 (r = –0.67, *p* < 0.001). When combined with the patient’s educational level, its predictive efficacy for MCI risk is improved (AUC = 0.85) [41]. Reduced miR-185-5p levels in neuron-specific exosomes are positively correlated with cortical Aβ positron emission tomography (Aβ-PET) burden (r = 0.61) [42]. Exosomal miR-185-5p regulates APP metabolism by binding to the 3^′^UTR to inhibit translation, thereby promoting APP protein expression and facilitating Aβ production and accumulation [62, 63]. miR-185-5p also targets the receptor for advanced glycation end products, inhibiting its expression and thereby reducing Aβ transport efficiency across the BBB into the brain [42]. In addition, miR-185-5p targets phosphoinositide-3-kinase regulatory subunit 3, activates the PI3K/Akt signaling pathway, inhibits neuronal apoptosis, and increases cell survival rates by approximately 30% [40]. Under specific stress conditions, AD neurons preferentially select pro-inflammatory miRNAs (e.g., miR-125b), which results in intracellular retention of miR-185-5p and subsequent uncontrolled APP translation [42]. miR-185-5p interacts with apolipoprotein E ε4, and its serum levels are markedly reduced in AD patients carrying this allele [62]. Therapeutically, engineered exosomes overexpressing miR-185-5p are capable of crossing the BBB and reducing hippocampal Aβ plaques in APP/PS1 mice by approximately 50% [42]. In summary, these findings underscore the potential of miR-185-5p as a therapeutic target for early intervention in AD patients.

miR-29b/c reportedly exerts dual regulatory effects by modulating amyloid-β production and the mitochondrial pathways in AD [43]. In AD, the CSF and serum exosomal miR-29b/c levels are considerably reduced, resulting in loss of inhibition on BACE1 and voltage-dependent anion channel 1 (VDAC1). This leads to increased BACE1 activity and enhanced Aβ production [64, 65]. The overexpression of VDAC1 reduces neuronal ATP synthesis by approximately 30%, markedly increases oxidative stress, and induces mitochondrial damage [66]. In clinical diagnostic studies, the CSF exosomal miR-29c levels were found to be negatively correlated with Aβ-PET burden (*r* = –0.74), with an AUC value of 0.92 for diagnosing SCD [18, 45]. In the treatment of an AD rat model, engineered MSC-derived exosomes overexpressing miR-29b were used to enhance their ability to cross the BBB. On the one hand, this approach reduces Aβ plaques by inhibiting the expression of BACE1; on the other hand, it upregulates SIRT1 expression, thereby improving synaptic plasticity [67].

Evidence from an AD mouse model has demonstratedd that miR-21-5p exerts bidirectional regulatory effects via the inflammation–metabolism axis. miR-21 expression is upregulated in the CSF of a subset of AD patients with MCI [44]. Contrarily, neuron-derived exosomal miR-21-5p is considerably downregulated, resulting in an elevated peroxisome proliferator-activated receptor alpha expression, disrupted lipid metabolism, increased mitochondrial reactive oxygen species production, and high neuronal apoptosis by approximately 40% [37, 44]. Conversely, MSC exosome delivery of miR-21-5p can inhibit the expression of PDCD4, thereby suppressing the activation of the NF-κB signaling pathway, which in turn reduces the production of neuroinflammatory factors TNF-α and IL-6 [38]. Additionally, miR-21-5p can also activate the PTEN/PI3K/Akt axis, promoting autophagy and facilitating the clearance of Aβ [45]. However, exosomal miR-21-5p in astrocytes is upregulated in the early stages of AD, potentially playing a pro-inflammatory role, whereas its downregulation in the late stages of AD is positively correlated with cognitive decline (*r* = 0.61) [37]. Thus, miR-21-5p exhibits stage-specific roles in the pathological progression of AD. In AD mouse models, specifically triple- transgenic mice, engineered exosomes loaded with miR-21-5p restored the synaptic density by approximately 80% and improved spatial memory [38]. In summary, miR-21 modulates Aβ oligomer-mediated toxicity in both *in vitro* and *in vivo* models. Although its specificity is limited [66], miR-21 is considered a multifunctional regulator in CNS disease progression, exerting both detrimental and beneficial effects [68].

## Current Challenges and Prospects

Exosome-mediated delivery of miRNA plays an important role in AD pathogenesis, as it targets multiple mRNAs. This brings new hope for AD diagnosis and treatment. However, this approach still faces a various severe challenges.

### Optimization and Standardization of Separation and Identification Techniques

The reliability and reproducibility of exosome research fundamentally depend on the rigor of isolation and characterization techniques [69]. Currently, mainstream separation techniques, including ultracentrifugation, size-exclusion chromatography, and polymer precipitation, are commonly used. However, exosomes from different biological samples have distinct characteristics in terms of recovery efficiency, purity, and ability to exclude non-exosomal vesicles, making data comparison across different studies difficult [70]. As found in the present study, miR-125b exhibits opposite trends in total plasma and neuron-derived exosome expressions. On the one hand, this may be due to the high heterogeneity of exosome populations in bodily fluids and the different preferences of isolation techniques for specific subpopulations [71]. On the other hand, it reveals that cell source specificity is the key to understanding the function of exosomal miRNAs. The signals measured in the total exosome-like samples represent a weighted average of signals from exosomes of diverse cellular origins, which may obscure or mask the specific signals that are most relevant to the underlying disease pathology [72].

Therefore, future research should shift from the analysis of total exosomes to the precise analysis of exosome subpopulations. By using techniques such as immunoaffinity capture based on the specific surface markers to actively enrich vesicles from particular cell types, it will be possible to effectively eliminate interference from irrelevant signals and to obtain more pathologically specific information.

### Complexity of Pathological Features

First, the limitations of single-target interventions are becoming increasingly apparent. The pathophysiological environment of AD is complex, involving dysregulation across multiple pathways. There is currently inadequate preclinical evidence to determine whether an intervention strategy targeting a single miRNA can achieve sufficient and lasting efficacy within such a complex network [73]. Additionally, exosomes themselves are complex functional carriers containing proteins, lipids, and nucleic acids. Exosomes contain various active components that may produce synergistic or antagonistic effects [74], making it difficult to attribute the observed overall efficacy entirely to a specific miRNA in therapeutic studies, thereby increasing the difficulty of the analysis of mechanism. Second, the functions of key molecules exhibit a high dependence on the body’s microenvironment and possess bidirectional regulatory characteristics. For example, miR-146a and miR-21-5p may play opposing roles, including pro-inflammatory versus anti-inflammatory or neuroprotective versusneurodamaging actions, depending on the disease stage and cell type releasing them. This indicates that their biological effects are not intrinsic but rather dependent on the state of the secreting cells, type of recipient cells, and overall pathological microenvironment [72]. This double-edged sword characteristic underscores the need for therapeutic strategies with precise spatiotemporal control capabilities. Finally, the safety of treatment and its long-term effects still require systematic evaluation. Although exosome carriers have naturally low immunogenicity, the therapeutic miRNAs they deliver may regulate extensive gene networks. Once inside the body, potential off-target effects and long-term interference with physiological signaling pathways are difficult to evaluate in the current animal models, posing potential risks for clinical translation [75]. In conclusion, it is further necessary to integrate multidimensional data, including transcriptomics and proteomics, to construct exosome-mediated gene regulatory networks, with the goal of systematically understanding their mechanisms of action. At the same time, exploring exosomes loaded with synergistic combinations of miRNAs or “smart” responsive exosome carriers may be a more promising approach to address the complex pathology of AD and achieve a safe and effective treatment.

### Construction and Validation of Clinical Translation Pathways

Existing evidence mostly comes from cross-sectional studies, which cannot clearly distinguish whether the observed miRNA changes are drivers of AD or secondary phenomena of disease progression. This limits their value as predictive biomarkers. Therefore, in this research field, there is an urgent need to conduct large-scale, multicenter, prospective cohort studies to monitor the progression from SCD and MCI to clinical AD over the long term [76]. Such studies should also evaluate the effectiveness of candidate exosomal miRNAs in predicting the rate of cognitive decline and disease stage progression, ultimately establishing their clinical decision thresholds for early screening and risk stratification. Regarding treatment, engineered exosomes offer broad potential as natural delivery carriers for therapeutic miRNAs, but their clinical translation remains considerably challenging. First, large-scale production and quality control are prerequisites for industrialization, yet the current methods face challenges in terms of yield, purity, and batch-to-batch consistency. Second, although exosomes have a natural tropism, their efficiency in *in vivo* targeted delivery—especially their ability to cross the BBB and accumulate in specific diseased neurons—still needs to be enhanced through advanced engineering techniques, such as surface ligand modification. In summary, exosomal miR-NAs, as an emerging therapeutic approach, show broad potential in AD treatment, but further in-depth research is still required.

## Conclusions

The present review demonstrated that specific exosomal miRNAs (miR-125b, miR-146a, miR-193b, miR-185-5p, miR-29b/c, and miR-21-5p) are robustly associated with AD progression and exhibit strong potential as diagnostic biomarkers. Their roles in regulating key pathways of AD (e.g., Aβ, Tau, and neuroinflammation) also highlight their therapeutic potential. Exosomal miRNA can serve as a diagnostic marker and therapeutic target for AD. However, large-scale sample validation is needed to support their clinical application.

## Availability of Data and Materials

The data are contained within the article.
